# Exploring determinants and strategies for implementing self-management support text messaging interventions in safety net clinics

**DOI:** 10.1017/cts.2022.503

**Published:** 2022-11-15

**Authors:** Lyndsay A. Nelson, McKenzie K. Roddy, Erin M. Bergner, Jesus Gonzalez, Chad Gentry, Lauren M. LeStourgeon, Sunil Kripalani, Pamela C. Hull, Lindsay S. Mayberry

**Affiliations:** 1 Department of Medicine, Vanderbilt University Medical Center, Nashville, TN, USA; 2 Center for Clinical Quality and Implementation Research, Vanderbilt University Medical Center, Nashville, TN, USA; 3 Quality Scholars Program, VA Tennessee Valley Healthcare System, US Department of Veteran Affairs, Nashville, TN, USA; 4 College of Medicine, University of Illinois at Chicago, Chicago, IL, USA; 5 Department of Pharmacy, College of Pharmacy and Health Sciences, Lipscomb University, Nashville, TN, USA; 6 Department of Behavioral Science, College of Medicine, University of Kentucky, Lexington, KY, USA; 7 Department of Biomedical Informatics, Vanderbilt University Medical Center, Nashville, TN, USA

**Keywords:** Mobile health, text messaging, implementation, behavioral intervention, type 2 diabetes, health disparities

## Abstract

**Background:**

Text message-delivered interventions for chronic disease self-management have potential to reduce health disparities, yet limited research has explored implementing these interventions into clinical care. We partnered with safety net clinics to evaluate a texting intervention for type 2 diabetes called REACH (Rapid Encouragement/Education And Communications for Health) in a randomized controlled trial. Following evaluation, we explored potential implementation determinants and recommended implementation strategies.

**Methods:**

We interviewed clinic staff (n = 14) and a subset of intervention participants (n = 36) to ask about REACH’s implementation potential. Using the Consolidated Framework for Implementation Research (CFIR) as an organizing framework, we coded transcripts and used thematic analysis to derive implementation barriers and facilitators. We integrated the CFIR-ERIC (Expert Recommendations for Implementing Change) Matching Tool, interview feedback, and the literature to recommend implementation strategies.

**Results:**

Implementation facilitators included low complexity, strong evidence and quality, available clinic resources, the need for a program to support diabetes self-management, and strong fit between REACH and both the clinics’ existing workflows and patients’ needs and resources. The barriers included REACH only being available in English, a lack of interoperability with electronic health record systems, patients’ concerns about diabetes stigma, limited funding, and high staff turnover. Categories of recommended implementation strategies included training and education, offering flexibility and adaptation, evaluating key processes, and securing funding.

**Conclusion:**

Text message-delivered interventions have strong potential for integration in low-resource settings as a supplement to care. Pursuing implementation can ensure patients benefit from these innovations and help close the research to practice gap.

## Background

Text message-delivered interventions have demonstrated efficacy in improving self-management across many chronic diseases [1–3], including type 2 diabetes [4,5], particularly among disadvantaged and racially diverse patients [3,6–8]. In contrast to Internet-based innovations which risk not benefiting all patients due to issues with access and digital literacy [9–11], text messaging can be accessed via any basic mobile phone and provides a digital interface most users are comfortable and familiar with [3]. However, despite ubiquity and evidence of efficacy [1,2], there are key knowledge gaps in integrating these interventions into clinical care [3].

Within the growing literature of studies examining implementation of technology-delivered interventions for chronic disease, few have evaluated text messaging programs specifically [3]. Relative to other forms of technology, these interventions carry unique considerations (e.g., cost, design, access) [3,12]. In addition, few studies have focused on safety net clinics which predominantly serve racial and ethnic minorities and persons who are underinsured. Finally, most research on the implementation of technology-delivered interventions has been limited to understanding barriers and facilitators to implementation [13–15]. More research is needed on leveraging these determinants to explore strategies for implementation.

Another critical limitation of studies examining implementation of technology-delivered interventions is that most have focused only on provider perspectives [13–15]. Excluding patients’ perspectives overlooks key determinants of successful implementation, such as factors that may impede patients from signing up for the intervention if integrated in their clinic. Because patients with lower socioeconomic status (SES) and racial and ethnic minorities are disproportionately affected by chronic disease and tend to experience worse outcomes, including these patients in implementation research is necessary to ensure their perspective is represented and strategies reflect their needs [16].

The current study addresses these gaps by examining barriers and facilitators to implementing a text message-delivered intervention for type 2 diabetes in safety net clinics and identifying appropriate implementation strategies. The intervention, Rapid Education/Encouragement And Communications for Health (REACH), includes interactive and tailored text messages supporting diabetes self-management. REACH was evaluated in a pragmatic, 15-month randomized controlled trial (RCT) using a hybrid type 1 effectiveness-implementation design [17–19]. The primary goal of the trial was to evaluate effectiveness [6]. This study describes the secondary goal to understand REACH’s implementation potential based on interviews with clinic staff and intervention participants.

## Methods

### Study Participants

The REACH intervention and trial outcomes have been reported in detail [6,17,18]. In a racially and socioeconomically diverse sample (n = 506), participants assigned to REACH had a hemoglobin A1c reduction of 0.74% among patients with baseline A1c ≥ 8.5% and reduction of 1.00% among patients with baseline A1c ≥ 10% compared to enhanced usual care (ps < 0.05). We found similar patterns of effects across validated self-report measures (e.g., self-efficacy, medication adherence, dietary behavior, and physical activity) [6]. This study focuses on understanding REACH’s implementation potential in safety net clinics, specifically; therefore, we interviewed patients and clinic staff from our five safety net clinic partners.

### Procedures

For patient interviews, we invited a subset of intervention participants using purposive sampling that sought balance on age, sex, race, education, income, clinic site, and owning a basic phone versus smartphone. Patient interviews were conducted in-person or over the phone and took approximately 20 min. For clinic staff interviews, we invited staff who served in multiple roles throughout the clinic using convenience sampling. Staff interviews were conducted in-person and took approximately 30 min. All interviewees completed informed consent and were paid $40. All interviews were audio recorded and transcribed verbatim. The Vanderbilt Institutional Review Board approved all study procedures.

### The Intervention

REACH is an automated, tailored, and theory-based text message-delivered intervention designed to improve adherence to diabetes medications. Participants receive three main types of text messages: self-care promotion one-way texts, interactive texts about medication adherence, and adherence feedback texts providing weekly feedback and encouragement based on responses to the interactive texts. The self-care promotion texts address nonmedication self-care behaviors (e.g., diet, exercise, or self-monitoring of blood glucose) and are tailored to medication adherence in two ways: patient’s prescribed regimen and barriers to adherence [20].

### Interview Guides

Interview questions were developed using the Consolidated Framework for Implementation Research (CFIR) [21]. The CFIR specifies 39 constructs associated with effective implementation arranged across 5 domains: the inner setting, outer setting, implementation process, characteristics of the individuals, and characteristics of the intervention [21] (Table 1). We consulted with experts in implementation science to review the CFIR constructs and select those most relevant to our proposed implementation. For clinic staff interviews, specifically, we identified five key processes integral to implementing a text messaging intervention in clinical care and presented these during the interview (Table 2). We asked clinic staff their thoughts and opinions about how each process might be integrated in the clinic. This was intended to highlight the primary difference between the RCT and the proposed implementation – primarily that clinic staff rather than research staff would now be responsible for these processes. Both the patient and staff interview guide are available in supplemental materials. We tagged each interview question with the respective CFIR domain and construct it was designed to address. All 5 CFIR domains were represented across each interview guide.

**Table 1.** Consolidated Framework for Implementation Research (CFIR) domains, definitions, and constructs

CFIR Domain	Definition	Constructs
Intervention Characteristics	Features of an intervention itself that may influence implementation	Intervention source, Evidence quality and strength, Relative advantage, Adaptability, Trialability, Complexity, Design quality and presentation, Cost
Outer Setting	Features of the external context or environment that might influence implementation	Patient needs and resources, Cosmopolitanism, Peer pressure, External policies and incentives
Inner Setting	Features of the implementing organization that might influence implementation	Structural characteristics, Networks and communications, Culture, Implementation climate, Tension for change, Relative priority, Organizational incentives and rewards, Goals and feedback, Learning climate, Readiness for implementation, Leadership engagement, Available resources, Access to knowledge and information
Characteristics of Individuals	Characteristics of the individuals involved that might influence implementation	Knowledge and beliefs about the intervention, Self-efficacy, Individual stage of change, Individual identification within the organization, Other personal attributes
Process	Activities or approaches that might influence implementation	Planning, Engaging, Opinion leaders, Formally appointed internal implementation leaders, Champions, External change agents, Executing, Reflecting and evaluating

**Table 2.** Key processes for implementing a text messaging intervention for self-management support in clinical care

Process	Description	Additional details/ considerations
Identification	Identify and inform eligible patients about program	Advertise program in clinic and/or reach out to patients (e.g., text, email, mail) about availability of program
Sign-up/Enrollment	Collect information from patients to begin program and tailor content and timing	Collect information as part of a brief survey that patients complete on their own or as part of a clinic visit (e.g., kiosk, tablet, or paper/pencil in the waiting or exam room) with or without assistance from staff
Retailoring	Collect information from patients approximately every 3 months for updating content	Monitor who is currently enrolled in program and when they are due for retailoring; then proceed with one of the options for Sign-up/Enrollment
Integrating	Integrate data collected as part of program into clinical care	View text responses before clinic visit and use to make recommendations (e.g., education and/or medication changes based on barriers to adherence and adherence rates); Monitor text responses to identify concerning responses and take appropriate action (e.g., if a patient reports non-adherence for 1 week; if a patient reports needing a refill)
Troubleshooting	Report and resolve technical issues to the technology company	Report issues with text message receipt/delivery and track progress with resolution

### Analyses

We used R version 4.1.1 (2021–08–10), NVivo 12, and Microsoft Excel for analyses. Thematic analysis was used to identify themes in the transcripts [22–24]. Using an open coding approach (inductive approach), we first identified themes and then classified them as barriers and facilitators. Next, we mapped CFIR constructs onto the coded data (deductive approach) [22–24]. Codebooks were developed for patient and staff interviews based on both initial open coding of transcripts, the CFIR-informed interview guide, and definitions of CFIR constructs. Two research staff coded a subset of interviews to determine inter-rater reliability, and once established, coded interviews independently. Then, author EMB reviewed coded data to map identified themes to the CFIR constructs as barriers or facilitators. Coders did not participate in the interview guide development and therefore had no preconceived notion of which CFIR constructs interview questions were supposed to query, reducing the risk of confirmation bias.

### Implementation Strategies

After coding the interviews, we integrated sources including the CFIR-Expert Recommendations for Implementing Change (ERIC) Strategy Matching Tool, interview feedback, and relevant literature to guide the selection of potential implementation strategies [15,25–27]. This process involved three team members, one of whom has advanced training in implementation science. First, we referenced the CFIR-ERIC tool to explore categories of implementation strategies that could be appropriate for addressing our identified barriers [25]. The CFIR-ERIC Matching Tool (publicly available on www.cfirguide.org) allows users to specify CFIR-based barriers and then generates a prioritized list of potential implementation strategies based on the endorsements of experts who were systematically queried during tool development [25,28]. Next, we synthesized the CFIR-ERIC tool results with suggestions for strategies provided in our interviews and strategies cited in the literature to generate more detailed recommendations. We organized recommendations by clusters of ERIC strategies [29].

## Results

### Patient Participants

Of the 46 patient participants invited to complete a follow-up interview, 36 (78%) did so (Table 3). Interviewed participants’ characteristics were similar to those of non-interviewed participants also recruited from safety net clinics (n = 180) [6]. Interviewed participants’ average age was 51.5 ± 11.0 years, 56% were female, 67% were non-White, 44% had a high school degree or less, and 53% had annual household incomes less than $25K. Average HbA1c was 9.0% ± 1.9% and 47% were prescribed insulin.

**Table 3.** Patient participant characteristics

M ± SD or % (n)	Interviewed participants (n = 36)^a^	Non-interviewed participants (n = 180)^b^
Age, years	51.5 ± 11.0	52.9 ± 9.9
Gender, female	56 (20)	52 (94)
Race/Ethnicity		
Non-Hispanic White	33 (12)	43 (76)
Non-Hispanic Black	53 (19)	44 (79)
Hispanic	11 (4)	7 (13)
Other race, including multiracial	3 (1)	6 (10)
Education, years	13.2 ± 2.1	12.8 ± 2.9
≤ High school degree	44 (16)	59 (107)
Annual Income < $25,000 USD	53 (19)	69 (124)
Clinics		
Clinic 1	19 (7)	28 (51)
Clinic 2	8 (3)	8 (14)
Clinic 3	17 (6)	12 (21)
Clinic 4	56 (20)	47 (84)
Clinic 5^**c**^	0 (0)	6 (10)
Owns smartphone	69 (25)	77 (139)
Baseline HbA1c	9.0 ± 1.9	8.7 ± 2.0
Prescribed Insulin	47 (17)	48 (86)
Diabetes Duration, years	9.2 ± 6.4	9.2 ± 7.4

USD, United States dollar; HbA1c, hemoglobin A1c

a Of the interviewed participants, 2 did not report years of education, 8 did not report income.

b Of the non-interviewed participants, 2 did not report race, 3 did not report education, 15 did not report income, and 2 did not report smartphone ownership

c Patients recruited from Clinic 5 were invited to complete an interview but declined to participate.

### Clinic Staff Participants

Eighteen staff across the safety net clinic locations were approached and 14 agreed to complete an interview. Six (43%) were in clinical roles (e.g., physician, nurse), four (29%) were in administrator roles (e.g., chief operations officer, clinic manager), and four (29%) were in other staff roles (e.g., registered dietician, pharmacist, social worker). Staff are identified with a study ID when reporting quotations.

### Implementation Determinants

**Fig. 1.**
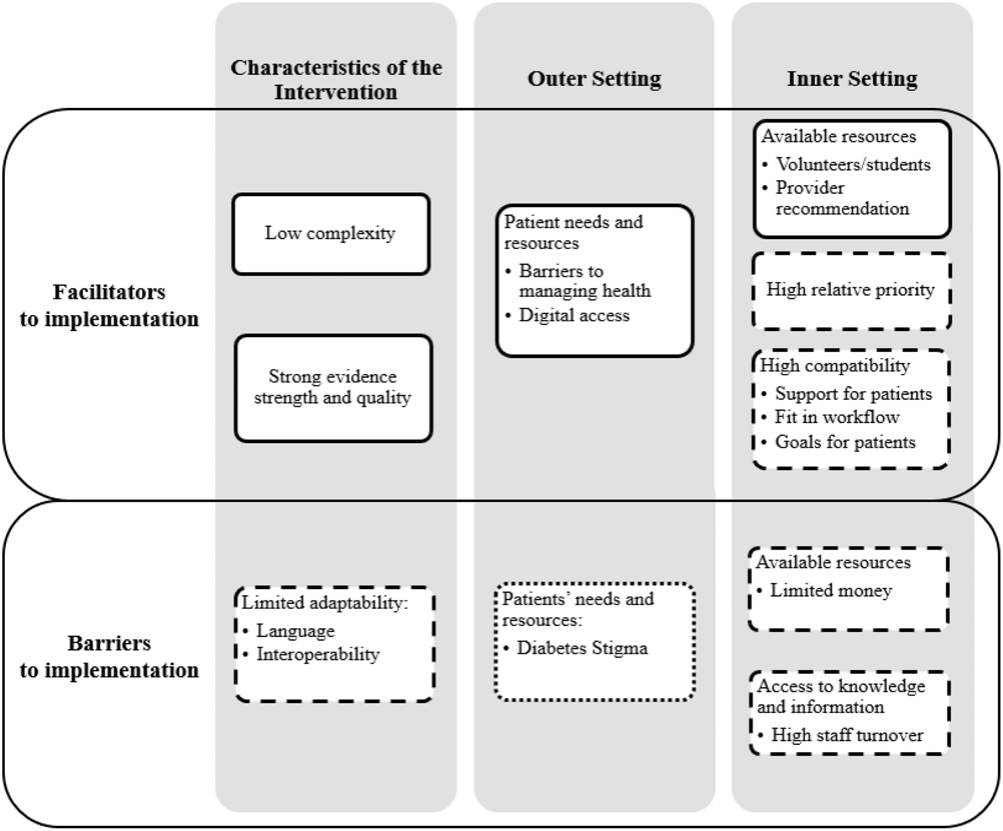
Facilitators and barriers to implementing REACH based on interviews with clinic staff and patients. Constructs with solid outlines were identified by clinic staff and patients; constructs with dashed lines were identified by clinic staff only; constructs with dotted lines were identified by patients only.

The implementation facilitators and barriers that emerged during the interviews are organized by the CFIR domains in Fig. 1. We collapsed findings from both the patient and staff interviews (using a similar format as Rogers et al. [30]). Although all five CFIR domains were represented on our interview guide, there were no identified facilitators or barriers that fell within the CFIR domains “characteristics of the individuals” or the “implementation process.” Due to our inductive/deductive approach to coding, some identified barriers and facilities clearly mapped onto the CFIR construct queried by the interview question, whereas others mapped onto a different construct.

### Facilitators

### Characteristics of the Intervention

#### Low complexity

Clinic staff shared the relative ease with which REACH could be implemented in clinics based on the simplicity of the program and its requirements. Staff appreciated that REACH was programmed to send automated text messages and only a few key processes would need to be integrated into the clinic setting to manage and oversee it. Staff highlighted how safety net clinics tend to be under-resourced and therefore minimal disruption to workflow is key.

*Everybody’s overworked, underpaid, and has too much on their plate… It would have to be integrated into a workflow that’s already there…And I do think that people who work at [safety net clinics] are all about, if you show me this works for my patients and its very minimal time upfront, we can do it. And [signing up patients] is almost something that can be done with a certified nursing assistant in the room…that would only take an extra five to ten minutes. (106, Clinician and Executive Director)*

Patients also described how REACH was easy to use which would facilitate patients signing up for and using the program if implemented. Patients liked that the program used text messages which they were familiar with and already used as part of their daily lives.

*I think it’s simple for people to be able to use. You have a cell phone, you get the text message, you look and see the message, and do whatever you would with the message, but it’s not intrusive. (Non-Hispanic, Black, Female, 46 years old)*

#### Strong evidence strength and quality

Both staff and patients described their confidence that REACH would improve patients’ self-management and glycemic control. Staff appreciated that REACH provided frequent, remote support in-between clinic visits, especially when patients may not receive regular care.

*It really engages our patients to take ownership of their disease. It’s so easy in primary care to just have the patients come in, and whether it be once a month …once every three months…it’s just easy for them to kind of go on autopilot. I feel like it was very beneficial for them to be engaged on a daily basis. (112, Pharmacist)*

During the RCT, clinic staff observed improvements in clinical outcomes among patients assigned to the intervention, as described by these clinicians:

*[REACH] really helped us with a lot of our [patients]. We have noticed an increase in control rates for our patients with diabetes. (109, Clinic Manager)*

*Some of our patients – in fact, I’m thinking of one in particular who was difficult to treat – when she got the one-on-one attention and frequent messages her A1c dropped tremendously. (108, Medical Director)*

Patients also felt that REACH was beneficial, with many describing increased accountability and awareness regarding their diabetes which led to better self-management.

*It made me want to be accountable for something that I know I needed to do…. And it made me proud to know that I could do it. Where I could respond with, “Yes, I took my medicine every day”…I felt like I was getting encouragement. (Non-Hispanic, Black, Female, 37 years old)*

*Just to tell a person, “You have to take this diabetic medicine.” – that’s not helping you. But the REACH program helped me to learn how to eat, how to take my medicine regularly. (Non-Hispanic, Black, Female, 60 years old)*

### Outer Setting

#### Patient needs and resources

Clinic staff were acutely aware of patients’ needs and felt REACH aligned highly with those needs. Safety net clinics predominantly serve underinsured patients who tend to have lower SES. Staff shared how their patients face many barriers to effectively managing their health:

*The patient population that we’re seeing is making choices whether to get their medications filled or to buy groceries. (112, Pharmacist)*

*I think a lot of our patients, if they do have insurance, there’s that [issue of] transportation. They can’t get in here all the time and get their blood drawn (113, Social Worker)*

Staff expressed how REACH would be well-suited to meet patients’ needs and resources because it requires little technological expertise and is delivered via cell phones which patients already have.

*I think a lot of high-tech interventions are not available to our patients, but a text message service is super easy for them. And we have very few patients that don’t have constant access to a cell phone. They may not answer when we call, but they have [a cell phone] and they’re getting a text message (107, Clinician and Executive Director)*

In addition, staff appreciated that the program provided patients with support and encouragement when they may be struggling to manage their health and other competing demands:

*I think they need the positive feedback; they need the encouragement… They like us being involved in their life and caring, and I think that this is just an extension of, “oh, these people care about me.” (102, Registered Dietician)*

Similarly, patients described how they were often juggling many different responsibilities including taking care of family members and working multiple jobs which could be very stressful. Receiving the REACH text messages fit well with their daily routines and helped motivate them:

*I know that a lot of people may think that they’re too busy…certainly the cell phone texting plan is such an easy thing. It doesn’t require any effort on your part. It just happens. (Non-Hispanic, White, Male, 59 years old).*

*For me, [I] get busy and the work that I do is stressful. So, sometimes if you see [a text], “Good job, you did it!”…that’s good for your self-care. I think it’s positive reinforcement. And when things are very stressful in the world today, to have something say, “You did a good job,” that’s an incentive to keep on doing better, exercise more, continue to make good food choices. (Non-Hispanic, Black, Female, 46 years old)*

### Inner Setting

#### Available resources

Clinic staff mentioned how different positions within the clinic could share managing the key processes for implementation. Several staff specifically said that volunteers and students could take on these roles and facilitate implementation.

*We have multiple providers going in [to meet with a patient] in a single visit, you know. So, it could be a social worker going in, it could be the pharmacist, the nurse practitioner, and all the student representatives in there. So, I think we have a lot of able-bodied people to help facilitate [informing and signing up patients] (112, Pharmacist)*

*I would say a volunteer coordinator could help with [monitoring text responses and communicating technical issues] for sure if they’re tech savvy. (102, Registered Dietician)*

Patients also mentioned several aspects of the clinic setting which would facilitate REACH’s implementation. First, patients shared how it would be important to advertise the program in the clinics, either using signage or a kiosk in the waiting room, and having front desk personnel mention it when patients checked in. Patients also shared how their providers’ involvement in the program would be critical to patient sign-up.

*Diabetes is a personal thing…a lot of people are very shy and intimidated about talking about it with anybody they don’t know…I would be more willing to talk to the doctor [about REACH] if he…kind of lay it out for you. Or, you know, even the nurses. They come in, take your temperature, do your blood pressure, all that kind of stuff, ask you what’s going on. You know, and that’s a chance for them to present the program, as well. (Hispanic, White, Male, 44 years old)*

#### High relative priority

A common theme across the staff interviews was the need for a program to help improve medication adherence. Staff described how many of their patients struggled with regularly taking their medications and the impact of non-adherence on their health.

*Yeah, I think [medication adherence is] a huge problem. And I’ve spoken about this before in different venues, but I tell people that you can make the best diagnosis in the world and write the best medicine in the world, but if people don’t take it, it doesn’t do them a lick of good. (101, Chief Medical Officer)*

*I’m very concerned [about medication adherence] just because realistically, we talk about lifestyle changes, but without intensive programs to work on lifestyle changes and constant motivation and all that kind of stuff it just doesn’t cut it. We don’t have a good way to make that work within our clinic structure. (106, Clinician and Executive director)*

#### Compatibility

Compatibility is defined as the degree of fit between meaning and values attached to the intervention by the individuals involved, how those align with individuals’ values and perceived needs, and how the intervention fits with existing workflows and systems [21]. As noted in the aforementioned themes, compatibility was evident concerning patients’ needs and resources, available clinic resources, and relative priority. Generally, there was a strong match between the REACH program and fulfilling patients’ needs (i.e., support for a prevalent health issue and via text messages which patients felt at ease with). Staff shared how REACH processes could be executed by staff within their roles and would fit with existing workflows.

Unique from themes previously identified was compatibility between REACH and clinic goals to offer diabetes programs to their patients. This clinic manager specifically appreciated that REACH’s content and functionality were already developed, and its efficacy had been tested in the RCT, such that it was ready to be implemented in the clinic:

*Us establishing and creating a program for diabetes is – we don’t have the bandwidth for that now. To partner with REACH, a program that is evidenced based now, created by our own evidence, and ready to implement on a larger scale. Writing a standard operating procedure, I think, is the next step for putting that in our [workflow]. [For example] someone with diabetes comes in, they get on REACH, we know that they’re gonna get this intervention to really, you know, lean into diabetes education, self-efficacy, and then if we add encouragement from us, like, “this is part of our bigger program to [change your] lifestyle. (109, Clinic Manager)*

### Implementation Barriers

### Characteristics of the Intervention

#### Limited adaptability

Two issues relevant to REACH’s adaptability were identified as barriers to implementation. First, clinic staff expressed concern that REACH was only available in English and not accessible to patients who spoke other languages.

*Since we have a large percentage of patients who …speak Spanish exclusively, that would be something that we would have to look at, too, just in order to reach most of our patients that we could. (106, Clinician and Executive Director)*

Second, staff expressed concern about interoperability. As REACH is currently designed, it does not integrate with clinics’ electronic health record (EHR) system. Rather, staff would need to access a separate dashboard for monitoring sent text messages and responses. When discussing this in the interviews, clinic staff felt it could be burdensome to open and navigate a separate system just for REACH.

*I mean ideally it would integrate into the EHR… I don’t know if it would ever integrate, but that’s going to be I think the biggest challenge, is how to integrate it and not creating another checklist for the provider to look at. (112, Pharmacist)*

*That’s where our frustration comes is when we have to document things in four different places in the same visit. You stop doing it because it’s so time-consuming because you’re putting it in all these different places. (110, Nurse Practitioner)*

### Outer Setting

#### Patients’ needs and resources

When asked what might prevent patients from signing up for REACH if the clinic offered it, several patient participants mentioned stigma related to diabetes. Responses did not specify how or why this might impede sign-up (e.g., not wanting to discuss their diagnosis upon sign-up or fearful others could see the texts on their phone), but this was the most commonly mentioned barrier:

*I think people wouldn’t want to do it because they [might] be embarrassed. Like, they don’t want people to know they got it. (Non-Hispanic, Black, Female, 26 years old)*

*I think they don’t like having diabetes, and they don’t like dealing with it. They just don’t want people to know they have diabetes, I guess…[like] it makes them a little weaker or something…stigmatization. (Non-Hispanic, White, Male, 56 years old)*

### Inner Setting

#### Available resources

During interviews, we explained that the main costs of implementing REACH would include a monthly fee to the technology company for maintaining the program and costs related to sending text messages. Clinic staff shared it would be challenging to pay for the program with their current budget but offered potential solutions for gathering funds to cover costs, including grants and donations from the community.

*I’ll be honest. I don’t think that I could get any more cost out of here. I would want to but, we would definitely partner [with other clinics] and say hey, do we wanna go ask [local community foundation] for money for this. (109, Clinic Manager)*

*We have a finance committee that applies for grants… I’m sure we could tap somebody on that committee to look for grants and apply for them…and we are pretty good at getting money from the community. So there’s always a possibility it’s within our budgetary means to find a way to support the program. (106, Clinician and Executive Director)*

*You might be able to get an outside source to do it. The way that I can see it being done is if the state looked at it and was like, “Oh, this program is awesome. It’s cheap. We should offer it on our state website and pay for it, and it’s free to every clinic.” Then that would be awesome. (110, Nurse Practitioner)*

#### Access to knowledge and information

Although many clinic staff shared that staff could help facilitate implementation, they also described how turnover in these positions (volunteers and students, especially) might make sustainability difficult. One clinic administrator referred to the importance of procedures in helping overcome issues related to turnover:

*That goes back to really having super well-defined standard operating procedures that you can sit down with somebody, because the issue with students is that they rotate. And so you may have somebody for a month…if you have a loosely defined procedure…you can’t clinically sustain that. (109, Clinic Manager)*

### Recommended Implementation Strategies

Based on the CFIR-ERIC tool results, the highest prioritized strategy for addressing limited adaptability was *promote adaptability* and for patient needs and resources was *obtain feedback from patients*. The highest prioritized strategy for available resources was *access new funding* and for access to knowledge and information was *conduct educational meetings*. After synthesizing these results with suggestions for strategies provided in our interviews and strategies cited in the literature, we produced four main categories of strategies. Below we outline these categories and provide examples of how strategies might be operationalized based on clinic needs and context.

#### Train and Educate Clinic Staff

To enhance implementation, strategies are needed to ensure clinic staff are knowledgeable of the intervention and its processes. Education/training should provide information about the intervention, how it is delivered, and relevant technical aspects. Ideally, staff training would be held both pre-implementation and during implementation to address ongoing questions and concerns [31,32]. To combat staff turnover as a barrier to implementation, we recommend creating materials that make it easy for new staff to learn about the program as part of their onboarding (e.g., toolkits delivered via manuals or videos). Identifying and preparing a clinic champion (e.g., to ensure staff remain up-to-date on training, maintain enthusiasm, and be the go-to person for questions) has been successful with implementing other technology-delivered interventions and can help facilitate training and education [15].

#### Offer Options for Adaptation and Tailoring

Tailoring is also essential for successful implementation. For example, key processes integral to implementing REACH (Table 1) can be completed through various options, tailored to clinic context and needs. Based on patient feedback, at least one option for identifying eligible patients should involve the provider. This may involve providers sending a letter to patients recommending the program or mentioning it as part of a clinic visit. Likewise, options for sign-up should include at least one method that is sensitive to the possibility of diabetes stigma (e.g., completing a survey privately in the exam room). Offering additional options (e.g., a kiosk in the waiting room) can provide increased convenience and efficiency to others for whom stigma is not a concern but will depend on clinic resources. Once options are identified, each process will need to be operationalized at the clinic level based on who is responsible for overseeing the process and how often. Lastly, based on clinic staff feedback, we learned that translating the REACH content to Spanish will make it accessible to more patients although this adaptation may require additional evaluation with Spanish speakers before implementation [33].

#### Use Evaluative and Iterative Strategies

Strategies designed to evaluate and monitor the key implementation processes can ensure those processes are adhered to. If an option for identification includes the provider recommending the program as part of a clinic visit, reminder prompts in the EHR can help facilitate. Likewise, audit and feedback could help encourage clinic staff to view and use the text message responses to inform care recommendations. In our interviews, we learned that clinic staff were concerned about the time and effort involved with navigating a separate system to view the REACH data. Although the most straightforward solution is to adapt REACH such that its data can be integrated with the EHR, this leads to larger issues. Safety net clinics, including the ones we partnered with for the RCT, tend to use a wide variety of EHR systems; therefore, investing in the interoperability of one will ultimately limit scalability as a whole. A more fruitful approach involves strategies that help overcome the underlying barrier of limited time and effort; this may include linking to the REACH dashboard directly from the EHR, involving other clinic staff (e.g., students and volunteers) to help monitor and triage the data, and/or developing quality monitoring systems.

#### Utilize Financial Strategies

During interviews, clinic staff mentioned several community organizations and funders who shared goals that aligned with REACH and could support implementation. Another strategy is to form a coalition of clinics implementing REACH to share costs owed to the technology company for maintaining the program.

## Discussion

Text messaging offers a ubiquitous, low-cost, and effective platform for improving chronic disease self-management and reducing health disparities, yet little is known about how to implement texting interventions in clinical care. Through interviews with patients and clinic staff, we explored the implementation potential of a text message-delivered intervention for diabetes medication adherence (REACH) in safety net clinics. Using the CFIR as an organizing framework, we identified barriers and facilitators to implementation and then used these findings to recommend implementation strategies. Patients and clinic staff appreciated that REACH supported patients’ self-care efforts and its ease of access; however, clinic staff were concerned about language limitations, funding, and staff turnover. Patients’ primary perceived barrier to other patients signing up for REACH was diabetes stigma. Key strategies for successful implementation include training and education, offering flexibility and adaptation, evaluating key processes, and securing funding.

Similar implementation determinants were identified in a study that examined the implementation of a text message-delivered intervention for insulin titration in safety net clinics (Mobile Insulin Titration Intervention [MITI]) [30]. In both REACH and MITI, facilitators were more readily identified than barriers. Clinic staff appreciated that these programs were relatively simple to integrate and aligned with their goals for improving patients’ health. Likewise, patients appreciated that the programs fit well with their regular routines, and attending to the text messages required little effort [30]. Both studies also identified a key barrier that the programs were only available in certain languages and would limit access to all patients. A key difference between the studies is that MITI assessed barriers and facilitators following an actual implementation, whereas we assessed these factors as part of a proposed implementation. As a result, the MITI study identified more barriers relevant to the CFIR domain of process (e.g., team nurses were not always available to enroll patients) which are difficult to ascertain without pursuing an actual implementation. In contrast to MITI, our study identified patients’ diabetes stigma as an implementation barrier. Half (52%) of patients with type 2 diabetes report feeling stigmatized [34] and disease-related stigma has been identified as a barrier to patients signing up for health services in other contexts including HIV and mental health [35,36]. Engaging patients to develop strategies sensitive to stigma can help mitigate this barrier [36].

We used an integrated approach to propose strategies for implementing REACH that involved multiple sources. First, we consulted the CFIR-ERIC Matching Tool and then combined those results with specific strategies mentioned in our interviews. We also referenced the literature to inform our recommendations [15,25–27]. Finally, we applied our strategies to the main clusters of ERIC implementation strategies [29]. Limited research has reported on the development of implementation strategies for technology-delivered interventions in diabetes [15]; however, in one notable exception, Ross et al. detailed an approach to their strategy selection for a digital self-management program for type 2 diabetes [26]. They also used a multi-step approach including a literature review to identify determinants of implementation, stakeholder engagement, and applying the Cochrane Effective Practice and Organisation Care taxonomy of implementation strategies [37]. Identified strategies included educational meetings and materials, local opinion leaders, audit and feedback, and continuous quality improvement [26]. Knapp et al. (2022) provide another useful method of how to meaningfully engage stakeholders to identify and specify implementation strategies [38].

There are several limitations to acknowledge. We used convenience sampling for the staff interviews by talking to staff we interfaced with during the trial and who they recommended we speak to. Although our final sample included staff in varied roles, the sampling approach may have biased representation of those interviewed. Further, in respect to both our staff and patient interviews, social desirability bias may have impacted responses. We summarized themes identified across all clinics to make general conclusions about factors that would impact implementation in these types of settings; however, additional work is needed to identify those factors which may be unique to certain clinics. Likewise, our process for developing strategies served only as a preliminary exploration. Our goal in using the interview data to recommend strategies was to provide a preliminary look at strategies that could address the barriers and facilitators identified across these diverse safety net clinics. While we hope the categories and examples serve as a starting point for others pursuing similar work, additional work with the clinics is a logical and necessary next step to delineate and tailor the strategies if pursuing an implementation study [38]. The results of the CFIR-ERIC tool should be interpreted with some caution due to wide heterogeneity in the experts’ endorsements of which strategies would best address barriers as part of the tool’s development [25]. Finally, the study findings were based on data from five safety net clinics in Nashville, TN; therefore, results may not be generalized to clinics in other regions.

## Conclusions

The evidence-practice gap is increasingly urgent concerning text messaging interventions for chronic disease self-management which have shown efficacy among vulnerable and disadvantaged persons with type 2 diabetes. Text messaging is a technology uniquely poised to reach those most in need to help reduce health disparities and require processes that can be feasibly integrated in low-resource settings. Pursuing how to best implement mHealth interventions into clinical care will help ensure the wider population of patients with type 2 diabetes can benefit now and in the future.

## Supplementary material

For supplementary material accompanying this paper visit https://doi.org/10.1017/cts.2022.503. click here to view supplementary material
