# Supplementary material for: Exploring determinants and strategies for implementing self-management support text messaging interventions in safety net clinics
Source: J Clin Transl Sci. 2022 Nov 15;6(1):e126. doi: 10.1017/cts.2022.503 (PMC9794969; doi:10.1017/cts.2022.503)
Supplement: Supplementary file 1 [file S2059866122005039sup001.docx]

**Supplemental Material**

**Clinic Administration/ Providers/Key Staff Interview Guide**

*[Interviews will be semi-structured, so interviewers may change the order of questions or ask follow-up questions to probe for additional information.]*

Introduction: “Over the past 2 years, your clinic has partnered with the REACH study to evaluate the effectiveness of the REACH cell phone program in improving patients’ diabetes management. The purpose of this interview is to gather information about how the REACH cell phone program could be implemented more widely into a clinical care setting and made available to patients, should the program be found to be effective. Before we get started with the interview, I’ll give you a brief overview of the REACH cell phone program.

Study participants who received the intervention, called REACH, received text messages at times of day they preferred which provided information and encouragement for diabetes self-care. Messages were tailored to their personal diabetes medication regimen and their barriers to medication adherence. They also received messages asking if they took their medicine with feedback on how adherent they were each week.

The other aspects of our partnership, including the surveys and the study A1c tests, were part of the study only and would not be a part of the program implementation moving forward. Please think about just the text messaging for these questions. Do you have any questions or want me to explain any of this more before we start the interview?” [*KSP answer any questions*]

“Let’s start the interview. I am now going to turn on the recorders.”

1. Overall, what do you think about the REACH cell phone program? (*CFIR Domain: Characteristics of Individuals; Construct: Knowledge and Beliefs about the Intervention*)
2. What, if anything, have you heard about participants’ experience with the REACH cell phone program? (*CFIR Domain: Outer Setting; Construct: Patients needs and resources*)
3. What are your clinic’s main goals for your patients with diabetes? (*CFIR Domain: Inner Setting; Construct: Compatibility*)
4. Does the REACH cell phone program meet the needs of your patients with diabetes? (*CFIR Domain: Outer Setting; Construct: Patient needs and resources*)
   - Why or Why not?
   - Which needs does it meet?
   - Which needs would you like to see it meet?
5. Does the REACH cell phone program align with other organizational goals? (*CFIR Domain: Inner Setting; Construct: Compatibility*)
   - If so, how?
6. Are there other diabetes programs already in your clinic or that you’d like to implement? (*CFIR Domain: Intervention Characteristics; Construct: Relative Advantage*)
   - If so, would these compete with REACH?
   - How do think a text messaging program might compare to these (or if none: other types of diabetes support interventions)?
7. Are you concerned about low rates of medication adherence among patients with diabetes at your clinic? (*CFIR Domain: Inner Setting; Construct: Relative Priority*)

- How does this concern compare to other concerns/priorities you have for patients at your clinic?

1. If our study finds that the REACH program is effective in reducing A1c levels, would you want the cell phone program to be available to your patients? (*CFIR Domain: Characteristics of the Intervention; Construct: Evidence strength and quality*)

- If no, why not?
- If yes:
  1. How could this cell phone program integrate into your other clinic processes? (*CFIR Domain: Inner Setting; Construct: Compatibility*)
  2. How should patients sign up for the program (e.g., Kiosk in waiting room, independently online, with their provider, with front desk staff)? (*CFIR Domain: Process; Construct: Engaging*)

1. If your clinic were to continue this program after this research is over, what aspects of your clinic environment could facilitate success? (*CFIR Domain: Inner Setting; Construct: Available resources*)
2. If your clinic were to continue this program after this research is over, what barriers do you foresee? (*CFIR Domain: Inner Setting; Construct: Available resources*)
   - Probing considerations (cost, staff time, technology, competing demands)
3. Do you think your clinic would pay to support offering REACH to patients if we find it reduces A1c? (*CFIR Domain: Inner Setting; Construct: Available resources*)
   - If no, why not?
   - If yes, what cost do you think would be reasonable? (e.g., per month or per year)
   - Are there financial structures (e.g., fee-for-service or pay-for-performance) or community financial donors that would influence implementing a program such as REACH?
   - If other clinics were interested in also implementing the program, would you be interested in sharing the maintenance fee for the program with these other clinics?
4. If the program were implemented, are there individuals in your clinic who you would feel comfortable appointing as a clinic champion to oversee implementation efforts? Would this individual have available bandwidth to oversee efforts or is it possible to have a shift in their responsibilities? (*CFIR Domain: Process; Construct: Champions*)
5. Is there anything else you’d like to tell me about the REACH program or about your experience with our team?

Refer to Table below outlining key processes involved in implementation:

1. What are your thoughts on the processes involved?
   1. What might be the best option for promoting/advertising the program? (*CFIR Domain: Process; Construct: Engaging*)
   2. What might be the best option to sign patients up for the program (e.g., at the clinic using kiosk or tablet, in the clinic waiting room or exam room, at home on their own time, or a combination of these options)? (*CFIR Domain: Process; Construct: Engaging*)
   3. Who in the clinic might be responsible for monitoring text message messages and/or resolving technical issues? (*CFIR Domain: Process; Construct: Engaging*)

“Thank you so much. This is the end of the interview. I am now going to turn off the recorders.” [*KSP turn off the recorders*]

| Key processes for implementing a text messaging intervention for self-management support in clinical care | | |
| --- | --- | --- |
| **Process** | **Description** | **Additional details/considerations** |
| **Identification** | Identify and inform eligible patients about program | Advertise program in clinic and/or reach out to patients (e.g., text, email, mail) about availability of program |
| **Sign-up/ Enrollment** | Collect information from patients to begin program and tailor content and timing | Collect information as part of a brief survey that patients complete on their own or as part of a clinic visit (e.g., kiosk, tablet, or paper/pencil in the waiting or exam room) with or without assistance from staff |
| **Retailoring** | Collect information from patients approximately every 3 months for updating content | Monitor who is currently enrolled in program and when they are due for retailoring; then proceed with one of the options for Sign-up/Enrollment |
| **Integrating** | Integrate data collected as part of program into clinical care | View text responses before clinic visit and use to make recommendations (e.g., education and/or medication changes based on barriers to adherence and adherence rates); Monitor text responses to identify concerning responses and take appropriate action (e.g., if a patient reports non-adherence for 1 week; if a patient reports needing a refill) |
| **Troubleshooting** | Report and resolve technical issues to the technology company | Report issues with text message receipt/delivery and track progress with resolution |

**Patient Participant Interview Guide**

*Interviews will be semi-structured, so interviewers may change the order of questions or ask follow-up questions to probe for additional information.*

“The purpose of this interview is for us to get your feedback about your experience using the REACH cell phone program during the study. Over the course of 15 months, you completed surveys and A1c tests, and used the cell phone program. For these questions, please only consider your experiences with the cell phone program. As a reminder, this included text messages with information, tips, and encouragement. You also got messages asking if you took your diabetes medicine and feedback on your progress. The information you share with us about your experience with the REACH cell phone program will help us understand what can be improved, and how it can be offered to other patients like you if we learn that the program helps patients better manage their diabetes. Do you have any questions before we start the interview? “[*KSP answer any questions*]

“Let’s start the interview. I am now going to turn on the recorders.” [*KSP turn on recorders*]

1. How would you describe the REACH cell phone program to your family or friends? (*CFIR Domain: Characteristics of Individuals; Construct: Knowledge and Beliefs about the Intervention*)
2. How would you describe the REACH cell phone program to someone like you who has diabetes? (*CFIR Domain: Characteristics of Individuals; Construct: Knowledge and Beliefs about the Intervention*)
3. Why did you decide to participate in REACH? *(CFIR Domain: Characteristics of the Individuals Involved; Construct: Other Personal Attributes)*
4. Did you find the REACH cell phone program helpful? (*CFIR Domain: Characteristics of the Intervention; Construct: Evidence strength and quality*)
   - If so, in what ways?
   - What was lacking? What would you have liked to see?
5. Compared to when you were receiving the cell phone program, has anything been different now that it has ended? *(CFIR Domain: Characteristics of the Individuals Involved; Construct: Other Personal Attributes)*
   - If so, please describe.
6. Did you have any challenges with the REACH cell phone program? *(CFIR Domain: Outer Setting; Constructs: Patient needs and resources)*
   - If so, what?
   - Did it get easier over time?
7. Do you think your clinic should offer the REACH cell phone program for their patients with diabetes? (*CFIR Domain: Characteristics of Individuals; Construct: Knowledge and Beliefs about the Intervention*)
   - Why or why not?
8. If your clinic offered the REACH cell phone program, would you sign up for the program again? *(CFIR Domain: Outer Setting; Constructs: Patient needs and resources)*
   - Why or why not?
   - Would you advise others to sign up? Why or why not?
9. How do you think people would prefer to sign up for the REACH cell phone program? (*CFIR Domain: Process; Construct: Engaging*)

[*If participant doesn’t understand*] For example, a kiosk in the waiting room, at home online, with their provider in the exam room, with front desk staff at the clinic?

1. What do you think would stop people from signing up for and using the REACH cell phone program? *(CFIR Domain: Outer Setting; Construct: Patient needs and resources)*
2. Do you think people would pay for the REACH cell phone program? How much per month do you think people would pay? *(CFIR Domain: Intervention Characteristics: Construct; Cost)*
3. Is there anything else you’d like to tell me about your experience in the REACH Study?

“Thank you so much. This is the end of the interview. I am now going to turn off the recorders.” [*KSP turn off the recorders*]
